# Assessment and Counseling Gaps Among Former Smokers Eligible for Lung Cancer Screening in US Adults: A Cross-Sectional Analysis of National Health and Nutrition Examination Surveys (NHANES), 2013–2018

**DOI:** 10.1007/s11606-022-07542-0

**Published:** 2022-04-26

**Authors:** Eve Angeline Hood-Medland, Melanie S. Dove, Elisa K. Tong

**Affiliations:** 1 Division of General Internal Medicine, Department of Internal Medicine, University of California, Davis, 4150 V St. #2400, Sacramento, CA 95817 USA; 2 Division of Health Policy and Management, Department of Public Health Sciences, University of California, Davis, Davis, USA

**Keywords:** tobacco, preventative medicine, lung cancer screening

## Abstract

**Background:**

Lung cancer screening (LCS) for former and current smokers requires that current smokers are counseled on tobacco treatment. In the USA, over 4 million former smokers are estimated to be eligible for LCS based on self-report for “not smoking now.” Tobacco use and exposure can be measured with the biomarker cotinine, a nicotine metabolite reflecting recent exposure.

**Objective:**

To examine predictors of tobacco use and exposure among self-reported former smokers eligible for LCS.

**Design:**

Cross-sectional study using the 2013–2018 National Health and Nutrition Examination Survey.

**Participants:**

Former smokers eligible for LCS (*n* = 472).

**Main Measures:**

Recent tobacco use was defined as reported tobacco use in the past 5 days or a cotinine level above the race/ethnic cut points for tobacco use. Recent tobacco exposure was measured among former smokers without recent tobacco use and defined as having a cotinine level above 0.05 ng/mL.

**Key Results:**

One in five former smokers eligible for LCS, totaling 1,416,485 adults, had recent tobacco use (21.4%, 95% confidence interval (CI) 15.8%, 27.0%), with about a third each using cigarettes, e-cigarettes, or other tobacco products. Among former smokers without recent tobacco use, over half (53.0%, 95% CI: 44.6%, 61.4%) had cotinine levels indicating recent tobacco exposure. Certain subgroups had higher percentages for tobacco use or exposure, especially those having quit within the past 3 years or living with a household smoker.

**Conclusions:**

Former smokers eligible for LCS should be asked about recent tobacco use and exposure and considered for cotinine testing. Nearly 1.5 million “former smokers” eligible for LCS may be current tobacco users who have been missed for counseling. The high percentage of “passive smokers” is at least double that of the general nonsmoking population. Counseling about the harms of tobacco use and exposure and resources is needed.

## INTRODUCTION

The US Preventive Services Task Force (USPSTF) recommends annual lung cancer screening (LCS) with low-dose computed tomography in eligible adults. As of 2021, the updated guidelines recommend screening for adults aged 50–80, who have a 20 pack-year smoking history, and currently smoke or have quit within the past 15 years.^1^ According to the 2010 National Health Interview Survey (NHIS), 14.3% of the US population met the previous criteria for LCS, half of which were former smokers.^2^ Extrapolating to the entire US population, 8.6 million Americans met the criteria for LCS, including 4.1 million former smokers.^2^

As part of annual LCS, smoking cessation interventions are strongly recommended for current smokers by the USPSTF and required by the Center for Medicare and Medicaid Services (CMS).^3^ A study by Howard et al. also recommends smoking cessation interventions for those eligible for screening and to include smoking cessation in clinical practice, as those advised by their physician are more likely to quit.^4^ After the initial 2014 USPSTF guidelines, smoking cessation interventions documented in the electronic medical record increased among eligible current smokers from 30.1 to 34.0%.^5, 6^ While recommendations are clear for current smokers who present for LCS,^7^ the guidelines for counseling former smokers are not well defined.

Prior studies have used cross-sectional statistical methods to inform data-driven understanding of screening trends, which can then inform care delivery.^8–10^ A study of current smokers using the National Health and Nutrition Examination Survey (NHANES) found good reliability of self-reported current smoking status and cotinine levels, which reflect tobacco exposure in the past few days, among adults eligible for LCS.^11^ However, less is known about the tobacco use and exposure among former smokers who are eligible for LCS. Understanding former smoker behavior is important because they may relapse (i.e., when a former smoker returns to smoking regularly^12^), especially within the first 6–12 months of quitting,^13^ and it can take smokers multiple attempts to quit for good.^14^ Also, while smoke exposure has substantially declined in the USA, 21.0% of nonsmoking adults aged 20 years and older, including former smokers, still had detectable cotinine levels in 2018, indicating exposure to tobacco smoke.^15^ The Surgeon General has concluded that there is no risk-free level of smoke exposure, and eliminating exposure is important not just for preventing lung cancer but more immediately for cardiovascular mortality.^16^ The purpose of this study is to identify high-risk former smokers who would benefit from targeted counseling at the time of LCS.

## METHODS

### Data Source

We used data from the 2013/2014 to 2017/2018 National Health and Nutrition Examination Survey (NHANES) (*n* = 28,061), as the tobacco questions were consistent during this time period.^17^ NHANES is a nationwide probability sample of the US civilian noninstitutionalized population conducted continuously and released in 2-year cycles.^18^

### Study Sample

**Figure 1:**
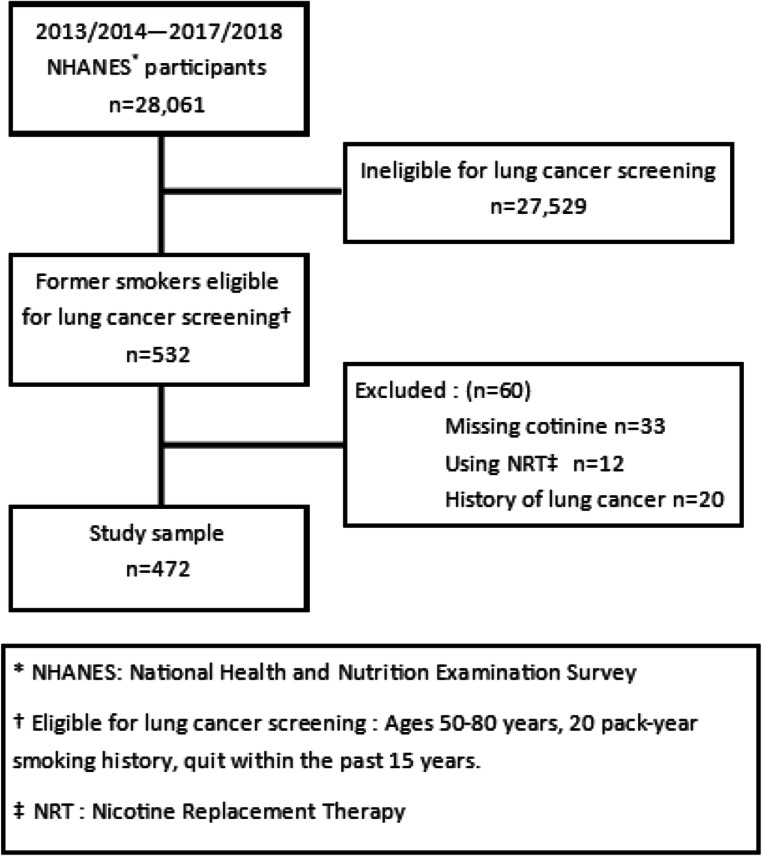
**Study sample eligible for lung cancer screening, NHANES 2013–2018.**

We included former smokers who were eligible for LCS (*n* = 532) (Fig. 1). Eligible former smokers who were missing cotinine (*n* = 33), using nicotine replacement therapy (*n* = 12), or had previously been diagnosed with lung cancer (*n* = 20) were excluded, for a final sample size of 472. This analysis of deidentified, secondary data was exempt from review as compliant with the policy of the University of California, Davis Institutional Review Board.

Former smokers were classified as those who had smoked at least 100 cigarettes in their life and responded, “Not at all” to the question “Do you now smoke cigarettes?”. Former smokers were eligible for LCS if they were 50–80 years old, had a 20 pack-year smoking history, and quit within the past 15 years. Pack-years were calculated by multiplying the calculated cigarette packs (number of cigarettes smoked per day divided by 20 cigarettes per pack) by the number of years smoked. Years since quitting was self-reported based on the following question “How long has it been since you quit smoking cigarettes?”. Years since quitting smoking were also categorized at 0–3, 4–6, and > 6 years, based on sample sizes. Age was top coded at 80, and therefore included adults 80 and older (*n* = 39) as eligible for LCS.

### Study Outcomes: Tobacco Use and Exposure

This study included two outcomes: recent tobacco use and exposure. Former smokers were classified as having recent tobacco *use* based on two sources: (1) self-reported use of any tobacco products in the past 5 days or (2) cotinine levels above the cut point for each racial/ethnic group (≥ 5.92 ng/mL (non-Hispanic Black), 4.85 ng/mL (non-Hispanic White), 0.84 ng/mL (Hispanic; this level reflects the largest subgroup being Mexican Americans^19^), and 3.08 ng/mL (all other).^20^ Former smokers without recent tobacco use were classified as having recent tobacco *exposure* if their cotinine levels were greater than 0.05 ng/mL. Although NHANES now has a lower limit of detection (0.011 ng/mL), we used 0.05 ng/mL for historical comparison with the general population.

### Tobacco History

Former smokers who recently used a tobacco product were further classified into using cigarettes, e-cigarettes, or another tobacco product (pipes, cigars, and smokeless tobacco were categorized together due to small sample sizes). We did not consider dual use of tobacco products due to small sample sizes. Former smokers were classified as living with a household smoker, if they responded with one or more to “How many people who live here smoke cigarettes, cigars, little cigars, pipes, water pipes, hookah, or any other tobacco product?” Former smokers were classified as being recently exposed to indoor smoke outside the home, if they reported that they spent time in an area (work, restaurant, bar, car, another home, or other indoor area) with someone else smoking in the past 7 days.

### Demographics and Medical Conditions

Self-reported demographic characteristics included age (50–64, 65–74, and 75–80 years), race/ethnicity (Hispanic, non-Hispanic White, non-Hispanic Black, and non-Hispanic Asian), gender (male, female), income (less than or equal to 100% of the federal poverty level), education (less than a high school graduate or GED and some college or a college graduate), married or living with a partner, type of health insurance (private, public [Medicare, Medicaid, or other], or uninsured), and survey cycle. Self-reported medical conditions included respiratory disease (emphysema, chronic bronchitis, or asthma), coronary heart disease or stroke, cancer, and diabetes. Depression was categorized as yes (mild, moderate, severe) or no based on the Patient Health Questionnaire (PHQ-9), which is a self-reported assessment based on DSM-IV signs and symptoms for depression.

### Data Analysis

Prevalence estimates of recent tobacco use and recent tobacco exposure among former smokers were estimated for each demographic characteristic, tobacco-related behavior, and medical condition. Differences in prevalence estimates were assessed using the chi-square test. Adjusted logistic regression analysis was used to examine the association between the characteristics and each outcome, adjusted for each characteristic (except for previous cancer). Sensitivity and specificity were calculated for self-reported recent tobacco use and cotinine levels with the racial/ethnic cut points for tobacco use.^20^ The sample size for the sensitivity and specificity analysis was 450 because 22 people were missing data on self-reported tobacco use in the past 5 days. To assess the stability of each prevalence estimate, relative standard errors (RSE) (standard error divided by estimate) were calculated. All analyses were weighted to account for differential sampling probabilities and response rates, and standard errors were adjusted for the survey design using survey-specific procedures in SAS 9.4 (SAS Institute, Cary NC).

## RESULTS

### Overview of Former Smokers Eligible for LCS

Among 50–80-year-old former smokers, 20.7% (95% CI 18.3%, 23.0%) or 6,937,000 adults (extrapolated to US population) were eligible for LCS (results not shown). As shown in Table 1, former smokers eligible for LCS were mostly non-Hispanic White (82.7%), male (67.1%), married or living with a partner (62.0%), with incomes above 100% federal poverty level (83.3%), and with either private health insurance (56.5%) or Medicare (25.6%). Over half were in the youngest age group (50–64 years) and had at least some college education. Less than half of former smokers (43.2%) had quit smoking less than 6 years ago. Approximately 1 in 5 reported living with a household smoker (22.4%) or having recent exposure in another indoor area (28.0%). Eligible former smokers reported a range of medical conditions: 26.5% had a respiratory condition (not shown: emphysema 11.6%, chronic bronchitis 14.6%, asthma 16.2%), 19.6% had coronary heart disease (CHD) or stroke, 16.7% had cancer (other than lung cancer), 30.2% had diabetes, and 27.1% had depression.

**Table 1:** Characteristics of Former Smokers Eligible for Lung Cancer Screening, NHANES 2013–2018 (*n* = 472)

		**Unweighted sample size**	**Weighted percent (95% CI)**
**Demographics**			
Age (years)	50–64	238	61.5 (55.5, 67.5)
	65–74	151	27.6 (22.3, 32.8)
	≥ 75	83	10.9 (8.3, 13.6)
Race/ethnicity	Hispanic	75	6.5 (4.5, 8.5)
	Non-Hispanic White	253	82.7 (79.0, 86.5)
	Non-Hispanic Black	97	1.2 (5.5, 10.5)
	Non-Hispanic Asian	33	0.56 (1.6, 3.9)
Male		332	67.1 (61.0, 73.1)
≤ 100% federal poverty level		128	16.7 (11.1, 22.3)
Education: at least some college or a college graduate		206	53.3 (46.5, 60.2)
Marital status: married or living with a partner		268	62.0 (54.9, 69.0)
Health insurance	Private	205	56.5 (48.3, 64.7)
	Medicare	162	25.6 (20.2, 31.0)
	Medicaid/other	60	10.4 (6.6, 14.2)
	Uninsured	44	7.5 (3.0, 12.0)
Survey cycle	2013/2014	155	32.1 (24.8, 39.5)
	2015/2016	165	36.5 (27.9, 45.1)
	2017/2018	152	31.4 (24.8, 38.0)
**Tobacco-related behaviors**			
Years since quitting	0–3	118	18.8 (13.3, 24.4)
	4–6	92	24.4 (16.6, 32.1)
	> 6	262	56.8 (50.8, 62.8)
Self-reported exposure to tobacco smoke	In the home	96	22.4 (15.6, 29.2)
	Indoor area outside the home	117	28.0 (21.3, 34.7)
**Medical conditions**			
Respiratory		139	26.5 (20.4, 32.6)
Coronary heart disease or stroke		102	19.6 (12.7, 26.6)
Cancer		69	16.7 (10.7, 22.7)
Depression		157	30.2 (24.6, 35.8)
Diabetes		154	27.1 (19.9, 34.4)

*CI* confidence intervals

### Former Smokers with Recent Tobacco Use

As shown in Table 2, about 1 in 5 (or 21.4%) former smokers eligible for LCS had recent use of a tobacco product, as defined by either self-reported use of tobacco in the past 5 days or cotinine levels indicating active tobacco use above racial/ethnic cut points. While 17.5% of former smokers reported recent use of a tobacco product in the past 5 days, 19.7% had cotinine levels indicating active tobacco use above racial/ethnic cut points (results not shown). There was high sensitivity (80.3%) and specificity (97.9%) between self-reported recent tobacco use and cotinine levels (results not shown).

**Table 2:** Association Between Characteristics and Recent Tobacco Use Among Former Smokers Eligible for Lung Cancer Screening, NHANES 2013–2018 (*n* = 472)

		Weighted percent (95% CI) with recent tobacco use*	***p*** **value**^†^	**Adjusted odds ratio (95% CI) (*n*** **= 419)**	***p*** **value**
**Total**		21.4 (15.8, 27.0)			
**Demographic**					
Age (years)	50–64	21.8 (13.1, 30.6)	0.73	0.93 (0.35, 2.53)	0.89
	65–74	22.6 (11.4, 33.9)		1.63 (0.52, 5.09)	0.39
	≥ 75	15.5 (8.0, 23.1)		ref	
Race/ethnicity	Hispanic	20.2 (8.5, 31.9)	0.28	0.84 (0.26, 2.70)	0.76
	Non-Hispanic White	21.5 (15.0, 27.9)		Ref	
	Non-Hispanic Black	30.8 (20.6, 41.1)		1.23 (0.57, 2.64)	0.59
	Non-Hispanic Asian	13.9 (0.77, 27.0)^‡^		1.08 (0.28, 4.10)	0.91
Gender	Male	24.9 (18.0, 31.8)	0.01	3.76 (1.54, 9.18)	0.005
	Female	14.1 (7.5, 20.8)		Ref	
Poverty	≤ 100% federal poverty level	26.1 (9.2, 43.0)^‡^	0.44	1.04 (0.32, 3.41)	0.94
	> 100% federal poverty level	20.4 (15.5, 25.3)		Ref	
Education	≤ High school or GED	21.9 (12.3, 31.6)	0.87	0.96 (0.30, 3.07)	0.95
	Some college or college grad	20.8 (13.1, 28.6)		Ref	
Married or living with a partner	Yes	20.5 (12.3, 28.7)	0.72	Ref	
	No	22.8 (13.8, 31.7)		2.12 (0.84, 5.37)	0.11
Health insurance	Private	22.0 (12.9, 31.2)	0.56	2.35 (0.75, 7.31)	0.14
	Medicare	16.8 (8.7, 24.9)		0.57 (0.17, 1.86)	0.34
	Medicaid/other	24.7 (9.6, 39.7)^‡^		1.50 (0.42, 5.38)	0.52
	Uninsured	29.1 (16.6, 41.6)		ref	
Survey cycle	2013/2014	23.4 (10.8, 36.0)	0.08	ref	
	2015/2016	13.6 (9.2, 18.0)		0.28 (0.10, 0.80)	0.02
	2017/2018	28.3 (16.5, 40.0)		1.42 (0.47, 4.27)	0.52
**Tobacco-related behaviors**					
Years since quitting	0–3	41.5 (28.7, 54.3)	< 0.001	5.89 (2.15, 16.1)	0.001
	4–6	27.3 (14.3, 40.3)		2.86 (0.96, 13.8)	0.06
	> 6	12.1 (6.6, 17.7)		Ref	
Household smoker	Yes	39.4 (21.7, 57.2)	0.01	5.77 (2.41, 13.8)	0.0002
	No	16.6 (10.3, 22.9)		Ref	
Exposed to indoor tobacco smoke	Yes	25.3 (13.5, 37.1)	0.39	0.89 (0.32, 2.46)	0.82
Outside the home	No	19.8 (13.6, 26.1)		Ref	
**Medical conditions**					
Respiratory	Yes	26.7 (16.4, 37.1)	0.20	1.46 (0.55, 3.87)	0.44
	No	19.4 (13.0, 25.9)		Ref	
Coronary heart disease or stroke	Yes	23.7 (8.9, 38.5)^‡^	0.68	1.25 (0.45, 3.46)	0.67
	No	20.8 (15.4, 26.2)		Ref	
Cancer	Yes	5.5 (1.3, 9.8)^‡^	< 0.001		
	No	24.6 (18.8, 30.3)			
Depression	Yes	25.5 (13.4, 37.5)	0.48	1.55 (0.51, 4.68)	0.43
	No	19.7 (11.4, 27.9)		Ref	
Diabetes	Yes	23.5 (14.5, 32.6)	0.52	1.01 (0.50, 2.04)	0.97
	No	20.3 (13.9, 26.8)		Ref	

Adjusted odds ratio adjusted for all variables listed in the table except for cancer

*CI* confidence intervals

*Recent tobacco use includes those with cotinine ≥ 5.92 ng/mL (non-Hispanic Black), 4.85 ng/mL (non-Hispanic White), 0.84 ng/mL (Hispanic), or 3.08 ng/mL (others), or self-reported tobacco use in the past 5 days

^†^*p* value from chi-square test

^‡^Relative standard error (RSE) between 30 and 50

Groups with a higher percentage of recent tobacco use than their counterparts include men, those who quit within the past 0–3 years, and those living with a household smoker (Table 2). In adjusted analysis, these differences remained statistically significant. Former smokers reporting having had a cancer diagnosis had a lower percentage of recent tobacco use than those who did not report cancer. Among the tobacco products used (not shown), about a third of former smokers used cigarettes (34.7%, 95% CI: 22.5%, 46.9%), less than a third used e-cigarettes (28.5%, 95% CI: 16.6%, 40.4%), and over a third used pipes, cigars, or smokeless tobacco (36.8%, 95% CI: 24.4%, 49.2%).

### Former Smokers with Recent Tobacco Exposure

Among former smokers without recent tobacco use, over half (53.0%) had cotinine levels above > 0.05 ng/mL, indicating recent tobacco exposure (Table 3). (Not shown, almost three-quarters (74.0%, 95% CI 65.9%, 82.0%) had any detectable cotinine > 0.011 ng/mL.) The majority (84.3%) of former smokers who reported living with a household smoker, and over two-thirds (71.1%) of former smokers who reported past-week exposure to indoor smoke outside the home, had cotinine levels > 0.05 ng/mL. Other groups with a higher percentage of tobacco exposure than their counterparts include those below the federal poverty level, those not married or living with a partner, and those who quit within the past.^7^ Hispanic former smokers had a lower percentage of tobacco exposure, compared with non-Hispanic White former smokers. These differences remained statistically significant in adjusted analysis.

**Table 3:** Association Between Characteristics and Recent Tobacco Exposure Among Former Smokers (Without Recent Tobacco Use) Eligible for Lung Cancer Screening, NHANES 2013–2018 (*n* = 375)

		Weighted percent (95% CI) with recent tobacco smoke exposure*	***p*** **value**^†^	**Adjusted odds ratio (95% CI) (*n*** **= 331)**	***p*** **value**
**Total**		53.0 (44.6, 61.4)			
**Demographic**					
Age (years)	50–64	54.7 (42.7, 66.6)	0.78	1.01 (0.40, 2.54)	0.98
	65–74	51.6 (38.3, 64.9)		1.26 (0.40, 3.98)	0.68
	≥ 75	48.0 (32.5, 63.5)		Ref	
Race/ethnicity	Hispanic	38.9 (25.4, 52.4)	0.01	0.17 (0.05, 0.57)	0.005
	Non-Hispanic White	50.2 (40.5, 59.9)		Ref	
	Non-Hispanic Black	72.1 (57.6, 86.5)		1.88 (0.76, 4.65)	0.17
	Non-Hispanic Asian	40.9 (23.0, 58.8)		1.10 (0.33, 3.64)	0.87
Gender	Male	56.3 (47.9, 64.8)	0.13	2.24 (0.92, 5.48)	0.08
	Female	47.2 (34.4, 60.0)		Ref	
Poverty	≤ 100% federal poverty level	78.4 (66.9, 89.9)	< 0.001	3.78 (1.67, 8.55)	0.002
	> 100% federal poverty level	48.3 (39.6, 57.0)		Ref	
Education	≤ High school or GED	56.1 (44.3, 67.9)	0.48	1.18 (0.56, 2.49)	0.67
	Some college or college grad	50.4 (38.7, 62.0)		Ref	
Married or living with a partner	Yes	46.3 (36.0, 56.6)	0.004	Ref	
	No	64.3 (54.2, 74.5)		3.07 (1.11, 8.48)	0.03
Health insurance	Private	48.6 (37.9, 59.2)	0.08	0.67 (0.15, 2.94)	0.58
	Medicare	53.5 (39.9, 67.0)		0.76 (0.19, 3.08)	0.70
	Medicaid/other	53.8 (34.1, 73.6)		0.51 (0.05, 5.34)	0.56
	Uninsured	81.4 (63.3, 99.4)‡		Ref	
Survey cycle	2013/2014	52.6 (38.6, 66.6)	0.81	Ref	
	2015/2016	50.4 (39.2, 61.7)		0.74 (0.31, 1.75)	0.48
	2017/2018	57.2 (38.2, 76.2)		0.91 (0.32, 2.63)	0.86
**Tobacco-related behaviors**					
Years since quitting	0–3	83.9 (72.2, 95.7)‡	0.0003	10.3 (3.75, 28.3)	< 0.001
	4–6	60.4 (42.4, 78.4)		2.12 (0.78, 5.77)	0.14
	> 6	43.6 (33.8, 53.4)		Ref	
Household smoker	Yes	84.3 (66.8, 100.0)§	0.001	5.33 (1.57, 18.0)	0.008
	No	46.2 (38.2, 54.1)		Ref	
Exposed to indoor tobacco smoke	Yes	71.1 (56.3, 85.9)	0.007	2.64 (1.08, 6.45)	0.03
Outside the home	No	46.5 (37.0, 56.0)		Ref	
**Medical conditions**					
Respiratory	Yes	57.1 (42.1, 72.1)	0.53	1.01 (0.36, 2.87)	0.98
	No	51.7 (42.2, 61.3)		Ref	
Coronary heart disease or stroke	Yes	48.5 (33.9, 63.1)	0.53	0.75 (0.27, 2.08)	0.57
	No	54.4 (44.1, 64.7)		Ref	
Cancer	Yes	54.5 (33.1, 75.9)	0.87		
	No	52.6 (43.2, 62.1)			
Depression	Yes	59.7 (47.6, 71.9)	0.17	Ref	
	No	49.5 (39.3, 59.6)		0.59 (0.30, 1.17)	0.13
Diabetes	Yes	47.8 (35.0, 60.7)	0.37	0.80 (0.35, 1.82)	0.59
	No	54.9 (44.7, 65.1)		Ref	

Adjusted odds ratio adjusted for all variables listed in the table except for cancer

*Recent tobacco smoke exposure includes those with cotinine > 0.05 ng/mL

†*p* value from chi-square test

^‡^Relative standard error (RSE) between 30 and 50

^§^Relative standard error > 50

## DISCUSSION

In a nationally representative study, there are high levels of recent tobacco use and exposure among former smokers eligible for LCS, which demonstrate a need for improved assessment and provider counseling. One in five survey respondents who identified as former smokers eligible for LCS had evidence of recent tobacco use which, extracted to the US population, represents approximately 1,416,485 adults. Over half of the remaining former smokers eligible for LCS had cotinine levels indicating tobacco exposure, which is double that of the general nonsmoking population.^21^ Providers can use this data to inform strategies that improve assessments and target former smokers for counseling on their continued risk for tobacco-related addiction, disease, and mortality, particularly for cardiovascular and respiratory disease.^16^

The NHANES question “do you now smoke cigarettes” may reflect how smoking status is assessed in current clinical practice. However, additional information about a patient’s current and former smoking status is needed. 1) For “Meaningful Use” requirements of electronic health records, the smoking status assessment categories of current (“every day,” “some day,” “heavy,” and “light”) and former smokers lack detailed definition.^22, 23^ Data collection about type of tobacco product used, last use, quit date, or exposure status are not required. 2) For tobacco assessment and counseling quality metrics, there is no defined timeframe for current or former tobacco status, nor is exposure status included.^23^, 24 3) For the LCS guidelines, tobacco status questions are focused on eligibility with total pack years of cigarette smoking and quitting within 15 years^7^ whereas also asking about recent tobacco use could better distinguish current and former smokers. Perhaps due to this lack of clarity, significant clinical discrepancies in documentation of smoking status by clinical providers in the electronic health record have been described, and health systems may benefit from updating LCS processes.^25^ Guidance for assessing tobacco use in clinical care should be clarified to minimize misclassification of current smokers and thus any missed opportunities for tobacco cessation counseling.

This study found that many respondents identified as “former smokers” had self-reported recent tobacco use or cotinine levels suggestive of active tobacco use. This may be due to response bias, tobacco exposure, or what a respondent considers “current tobacco use.” Asking about tobacco product use in the past month might be a preferable standard that aligns with documenting current “some day” smoking. Some former smokers may use tobacco irregularly, not meeting criteria for current use or “relapse.” However, “some day” smokers, even with just 6–10 cigarettes per month, still have higher mortality risks than never smokers.^26^ More information on current tobacco use by self-reported former smokers would provide opportunities for targeted counseling. A suggested improvement is to have patients submit self-reported responses to standardized questions.^27^

This study found that over half of former smokers without recent tobacco use had higher proportions of tobacco exposure than the general population, as measured by cotinine. This underscores the need for provider counseling that addresses the environment. Patients may be counseled that there is no risk-free level of smoke exposure and advised to make a smoke-free home rule, not just for the continued risk of developing lung cancer but more immediately for cardiovascular health.^16, 21^ There is growing evidence for family system interventions to support quitting.^28^ For example, a brief to moderate intensity educational intervention about smoke-free living with a smoker and household nonsmoker led to long-term quit rates similar to standard smoking cessation trials.^29^ Referral to evidence-based tobacco treatment resources such as quitlines can also be offered to family or household members who use tobacco.^30^ All states have access to a quitline, which provides free evidence-based counseling that doubles the chances of long-term quitting.^13^

Cotinine might be a useful adjunct to counseling for biomarker feedback and has been suggested in LCS guidelines as a possible tool for education and counseling.^31^ Biomarker feedback would be helpful for the 53% of former smokers who did not recently use tobacco and had biochemically validated exposure, as only 22.4% of all former smokers self-reported living with a smoker or having recent indoor exposure; measuring cotinine may initiate a discussion about identifying additional sources of exposure. Measuring cotinine in current tobacco users may have marginal benefit, as the reliability of self-reported tobacco use in the past 5 days was confirmed by the cotinine analyses in our study. We found a sensitivity and specificity of 80.3% and 97.9% among former smokers who recently used a tobacco product, which is slightly lower than 86.4% and 99.7% in a study^21^ that examined current smokers and cotinine using NHANES data over a 20-year period. If former smoker status is assessed by recent tobacco use instead of the question “do you now smoke cigarettes,” measuring cotinine levels may be best suited in former smokers with secondhand smoke exposure to guide conversations on risk. Unfortunately, the high sensitivity cotinine lab test may not be widely available in commercial laboratories. Future studies should assess whether including cotinine levels may change patient behavior and outcomes, and what the cost-effectiveness of this approach might be.

Two factors associated with having a higher percentage of both recent tobacco use or exposure were living with a household smoker or having recently quit within the past 0–3 years. This finding underscores the need for continued counseling among former smokers about the risk of relapse or ongoing nicotine addiction and any environmental or behavioral changes needed to eliminate exposure. Other subgroups with a higher percentage of exposure included those below the federal poverty level, and those not married or living with a partner. Low socioeconomic groups also have access barriers to LCS as previous studies have found lower rates of screening among the uninsured,^32, 33^ and not all Medicaid programs cover LCS.^34^ For racial/ethnic subgroups, only Hispanic former smokers had a lower risk of smoke exposure than non-Hispanic White former smokers. Our study used the 2021 guidelines which have lower age and pack-year requirements that help include more racial/ethnic subgroups.^34^ Identifying these higher-risk subgroups of former smokers may help future studies or guideline updates about targeted counseling for patients identified as former smokers.

Improving smoking cessation interventions for LCS is needed and ongoing. Although smoking cessation interventions documented in the electronic medical record increased after the 2013 USPSTF LCS guidelines, only 34% of adults eligible for LCS post-guidelines received any sort of smoking cessation intervention from their health care provider.^5^ The Optimizing Lung Screening (OaSiS) cluster randomized trial will evaluate strategies to implement smoking cessation interventions during LCS.^35^ A meta-analysis of smoking cessation interventions to use during LCS found that counseling and pharmacotherapy increased cessation at 12 months.^36^ Studies are also in effect to examine changing eligibility criteria to include risk prediction models to limit over-diagnosis.^37^ These efforts may be extended to examine strategies for former smokers eligible for LCS.

This study had several limitations. Self-reported smoking status may be subject to social desirability and reporting biases. Serum cotinine reflects only recent tobacco exposure over the past few days and other biomarkers may reflect longer exposure periods or distinguish those using nicotine-replacement products. NHANES assigns adults older than 80 years old an age value of 80, and our study may slightly overestimate the number of former smokers eligible for LCS (4.6 million compared with 4.1 million found using NHIS).^2^

## CONCLUSION

Former smokers eligible for LCS should be asked and counseled about recent tobacco use and exposure and considered for cotinine testing. Tobacco status assessment for former smokers should include questions about last tobacco use, type of tobacco product, household smokers, and indoor exposure. Certain subgroups are at higher risk for tobacco use or exposure, especially those having quit within the past 3 years or living with a household smoker. Data-driven counseling can target tobacco-related disease and mortality, steps for a healthier environment, and assistance for household tobacco users.
